# Supplementary material for: Transcriptome Analysis of Caco-2 Cells upon the Exposure of Mycotoxin Deoxynivalenol and Its Acetylated Derivatives
Source: Toxins (Basel). 2021 Feb 22;13(2):167. doi: 10.3390/toxins13020167 (PMC7927021; doi:10.3390/toxins13020167)
Supplement: Supplementary file 1 [file toxins-13-00167-s001.pdf]

Supplementary Materials: Transcriptome Analysis of Caco-2 Cells upon the Exposure of Mycotoxin Deoxynivalenol and Its Acetylated Derivatives

Yuyun He, Xiaoyao Yin, Jingjing Dong, Qing Yang, Yongning Wu and Zhiyong Gong

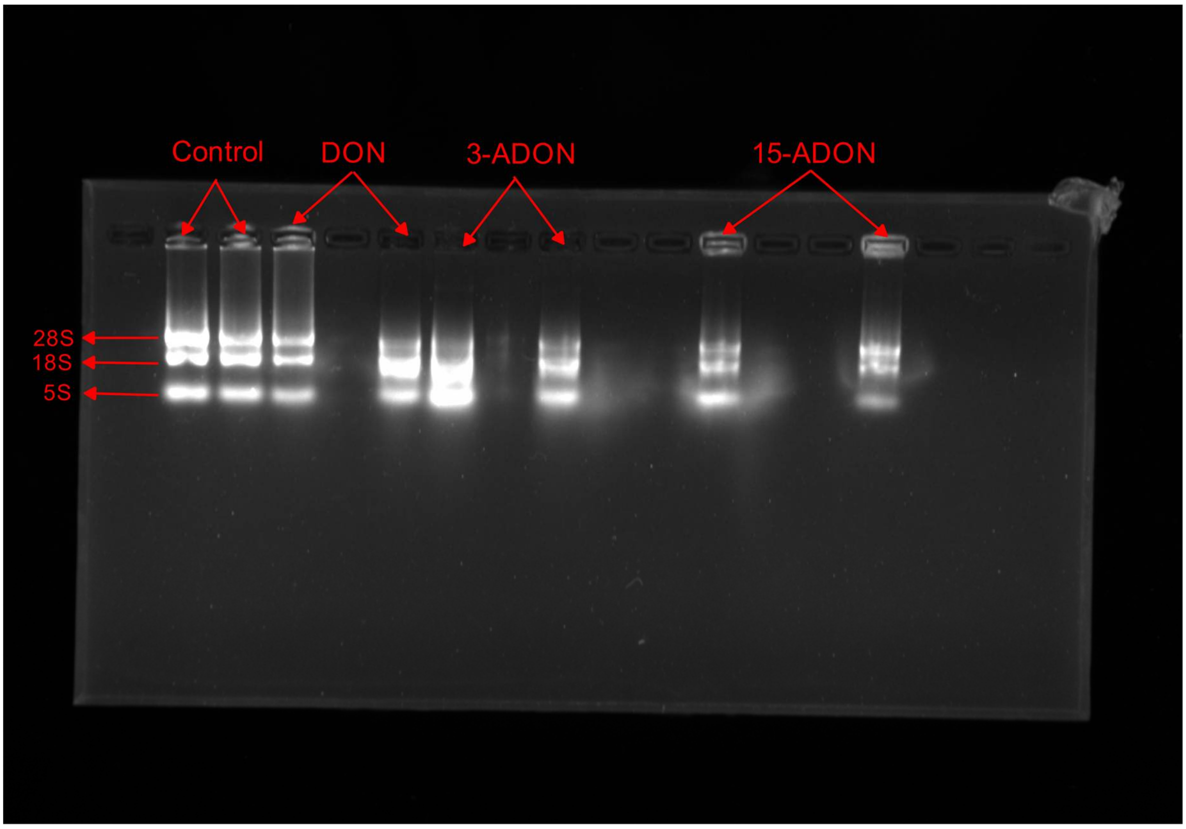

Figure S1. RNA degradation gels images.
